# The Roles of Moonlighting Nicotinamide Mononucleotide Adenylyl Transferases in Cell Physiology

**DOI:** 10.3390/ijms26189098

**Published:** 2025-09-18

**Authors:** Yi-Ching Lee, Su-Ju Lin

**Affiliations:** Department of Microbiology and Molecular Genetics, College of Biological Sciences, University of California, Davis, CA 95616, USA; ycclee@ucdavis.edu

**Keywords:** multi-functional enzymes, NMNAT, NAD^+^ metabolism, chaperones, neuroprotection

## Abstract

Nicotinamide adenine dinucleotide (NAD^+^) is an essential metabolite, and abnormal NAD^+^ metabolism has been linked to numerous human diseases. The nicotinamide mononucleotide adenylyl transferases (NMNATs) catalyze NAD^+^ production through both de novo and salvage pathways. NMNATs are multi-functional enzymes with NAD^+^ synthesis activity and chaperone activity. Interestingly, NMNATs are involved in neuroprotection, and whether these neuroprotective effects require NAD^+^ synthesis activity appears to vary depending on the context. Nevertheless, NMNATs can modulate cellular processes primarily through supporting NAD^+^ homeostasis. In this review, we discuss the roles of NMNATs in NAD^+^ homeostasis, their functional domains, and how their subcellular localizations influence the compartmentalized NAD^+^ pools. We present an integrative framework to help understand the diverse impacts of NMNATs in human diseases, with a focus on neurological disorders caused by different insults. To address knowledge gaps, we integrate the regulation of NMNATs in both human and model organisms. We also discuss the current understanding and limitations of NMNAT activators and inhibitors to help evaluate their translational significance as therapeutic targets for NAD^+^ modulation.

## 1. Introduction

Nicotinamide adenine dinucleotide (NAD^+^) is an essential factor involved in various metabolic reactions, where NAD^+^ functions as a hydride acceptor and its reduced form, NADH, functions as a hydride donor. In addition to its roles in oxidation and reduction reactions, NAD^+^ is also a substrate for NAD^+^-consuming enzymes, including sirtuins [1,2,3], poly (ADP-ribose) polymerases (PARPs) [4], CD38 [5], CD157 [6], and SARM1 [7]. Therefore, NAD^+^ is involved in a myriad of cellular processes, including gene regulation, DNA repair, immune response, and neurodegeneration. Abnormality in NAD^+^ homeostasis has been implicated in numerous age-associated disorders, and harnessing NAD^+^ metabolism is an emerging therapeutic strategy for the treatment and prevention of specific human diseases [8,9,10,11,12].

Enzymes involved in NAD^+^ metabolism are potential targets for NAD^+^ modulation. For instance, nicotinamide phosphoribosyl transferase (NAMPT) is a rate-limiting enzyme for mammalian NAD^+^ synthesis [13,14,15], and NAMPT inhibitors have been shown to reduce NAD^+^ levels as well as induce cell death in various cancers [16,17,18]. Nevertheless, NAMPT inhibitors display significant toxicity in phase I clinical trial [19,20]. Nicotinamide N-methyltransferase (NNMT) is another important factor that modulates NAD^+^ homeostasis, and inhibition of NNMT is a strategy to increase NAD^+^ production. NNMT methylates nicotinamide (NAM) using S-adenosylmethionine (SAM) as a methyl donor, forming 1-methylnicotinamide (MNAM) [21]. MNAM cannot enter the salvage pathway to synthesize NAD^+^; therefore, NNMT limits the salvage of NAM and down-regulates NAD^+^ synthesis. NNMT also alters the epigenetic landscape by limiting the availability of SAM [22], the primary methyl donor for histone methyltransferases. Studies of NNMT inhibitors have shown promise in treating metabolic disorders and cancers [23]. Nevertheless, it is important to note that NNMT and MNAM are involved in the protection of endothelial cells against various stresses [24,25,26,27]. Thus, the use of NNMT inhibitors should consider the potential side effects on the endothelium.

Targeting the nicotinamide mononucleotide adenylyl transferases (NMNATs) may represent another efficient strategy to modulate NAD^+^ metabolism due to their involvement in all NAD^+^ biosynthetic routes (to be discussed in Section 2, Overview of NAD^+^ Metabolism). Notably, NMNATs are also known for their neuroprotective effects, with numerous studies showing that overexpressing NMNATs can protect neurons in various models of neurodegenerative diseases [28,29,30,31,32], and the absence of NMNAT is shown to deteriorate neuronal integrity as well as trigger axon degeneration [33,34]. It appears that NMNATs may function beyond merely being NAD^+^ biosynthetic enzymes and that their chaperone activity may also protect cells from proteotoxicity [30,34,35,36,37]. This review aims to explore the diverse roles of NMNATs, with a specific focus on the contribution of NMNATs to neuroprotection, and to provide insights into whether the NAD^+^ synthesis activity and/or the chaperone activity of NMNATs are essential in neuroprotection. On the other hand, current knowledge on the regulation of human NMNATs is limited. Here, we also summarize studies of NMNAT regulation in model organisms to provide a comprehensive overview and to help address this knowledge gap. We also extensively outline the known compounds that may affect the expression level and enzyme activity of NMNATs. Understanding the complex NMNATs regulatory network and their physiological effects may provide new insights into NAD^+^ modulation as well as therapeutic strategies for human diseases.

## 2. Overview of NAD^+^ Metabolism

**Figure 1:**
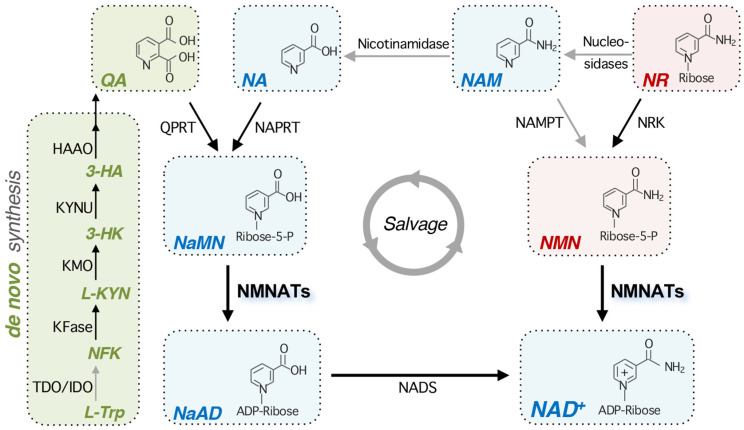
An overview of the NAD^+^ biosynthetic pathways. In eukaryotes, NAD^+^ can be synthesized de novo from the amino acid L-Trp. The conversion of L-Trp to NFK is rate-limiting and is catalyzed by TDO or IDO. After another four enzymatic reactions and one spontaneous cyclization, QA is produced and then converted to NaMN by QPRT. In the salvage pathways, NAD^+^ is maintained by the recycling and assimilation of NAD^+^ precursors NA, NAM, and NR into NAD^+^. NA is converted to NaMN by NAPRT. NMNATs then convert NaMN to NaAD, and subsequently, NADS catalyzes the amidation of NaAD into NAD^+^. NR is phosphorylated by NRK, producing NMN. NMNATs then convert NMN to NAD^+^. Note that NMNATs have dual substrate specificity for NMN and NaMN. NR is dynamically converted to NAM by several nucleosidases. In mammals, NAM can be converted to NMN by NAMPT. Although NAMPT is not found in bacteria and yeast, NAM can be converted to NA by the nicotinamidases (PncA/Pnc1) in these species. Mammalian cells do not possess any functional equivalent of nicotinamidase; however, in the gastrointestinal tract, the gut microbiome produces nicotinamidases and helps convert NAM into NA. Steps shown with gray arrows indicate that they may be missing in some organisms or are mediated by various species-specific enzymes. Abbreviations of NAD^+^ intermediates are italicized. *L*-*Trp*, L-tryptophan; *NFK*, N-formylkynurenine; *L-KYN*, L-kynurenine; *3-HK*,3-hydroxykynurenine; *3-HA*, 3-hydroxyanthranilic acid; *QA*, quinolinic acid; *NA*, nicotinic acid; *NaMN*, nicotinic acid mononucleotide; *NaAD*, deamido-NAD^+^; *NAM*, nicotinamide; *NR*, nicotinamide riboside; *NMN*, nicotinamide mononucleotide. Abbreviations of protein names are shown below. TDO, tryptophan 2,3-dioxygenase; IDO, indoleamine 2,3-dioxygenase; KFase, kynurenine formamidase; KMO, kynurenine 3-monooxygenase; KYNU, L-kynurenine hydrolase; HAAO, 3-hydroxyanthranilic acid 3,4-dioxygenase; QPRT, quinolinate phosphoribosyl transferase; NAPRT, nicotinate phosphoribosyl transferase; NMNAT, nicotinamide mononucleotide adenylyl transferase; NADS, NAD^+^ synthetase; NAMPT, nicotinamide phosphoribosyl transferase; NRK, NR kinase.

NAD^+^ homeostasis is maintained by balancing NAD^+^ synthesis and degradation. While NAD^+^-consuming enzymes [1,2,3,4,5,6,7] break down NAD^+^, NAD^+^ biosynthetic enzymes replenish the NAD^+^ pool through multiple pathways. The NAD^+^ pool is maintained by three biosynthesis pathways, which are largely conserved from bacteria to humans. In eukaryotes, cells can produce NAD^+^ through de novo synthesis of quinolinic acid (QA) from the amino acid L-tryptophan (also known as the kynurenine pathway or tryptophan degradation pathway) or through salvaging NAD^+^ precursors like nicotinic acid (NA), nicotinamide (NAM), and nicotinamide riboside (NR) (Figure 1). The de novo pathway produces QA through a series of enzymatic reactions. In yeast, these enzymes are named Bna (Biosynthesis of nicotinic acid) proteins which include Bna2 (TDO/IDO), Bna7 (KFase), Bna4 (KMO), Bna5 (KYNU), and Bna1 (HAAO). QA is then converted by the QA phosphoribosyl transferase (QPRT/Bna6) to nicotinic acid mononucleotide (NaMN), converging on the NA-NAM salvage pathway. Notably, several enzymatic reactions in the de novo pathway require molecular oxygen as a substrate; therefore, cells grown under anaerobic conditions rely on salvage pathways for NAD^+^ synthesis [38].

The salvage of different NAD^+^ precursors are interconnected, rendering flexibility for cells to produce NAD^+^. NAD^+^ synthesis using NA as a precursor is known as the Preiss-Handler pathway [39]. In yeast, this pathway includes the salvage of NAM, and is referred to as the NA-NAM salvage pathway. In the NA-NAM salvage pathway, NA is converted to NaMN via the nicotinate phosphoribosyl transferase (NAPRT). NaMN is then converted to nicotinic acid adenine dinucleotide (NaAD) via NMNATs [40,41]. Finally, NaAD is amidated to NAD^+^ via the NAD^+^ synthetase (NADS) [42]. The salvage of NAM is relatively less conserved in different species. In budding yeast, NAM is converted to NA through the nicotinamidase Pnc1. In mammals, NAM is mainly converted to nicotinamide mononucleotide (NMN) by nicotinamide phosphoribosyl transferase (NAMPT) [13,14,15]. While the nicotinamidase is absent in mammalian cells, it is reported that the gut microbiota help convert NAM to NA via the bacterial nicotinamidase PncA [43]. To be noted, NAD^+^-consuming enzymes break down NAD^+^ and produce NAM as a byproduct. The salvage of NAM is critical as it recycles NAM arising from NAD^+^-dependent signaling reactions and turns it back to NAD^+^. In the NR salvage pathway, NR can enter the NA-NAM salvage pathway when it is converted to NAM by several nucleosidases. In budding yeast, reported NR nucleosidases include Urh1, Meu1, and Pnp1 [44]. NR can also be converted to NMN by the NR kinase (NRK). Subsequently, NMN is adenylated to NAD^+^ by NMNATs. Moreover, NMNATs exhibit dual substrate specificity, recognizing both NMN and NaMN [40,45]. By transferring the adenylyl group from ATP to NMN and NaMN, NMNATs catalyze the production of the dinucleotides, NAD^+^ and NaAD, respectively. As a result, NMNATs are essential for NAD^+^ biosynthesis, as they function in both the de novo and salvage pathways.

## 3. Functional and Conserved Motifs in NMNATs

**Figure 2:**
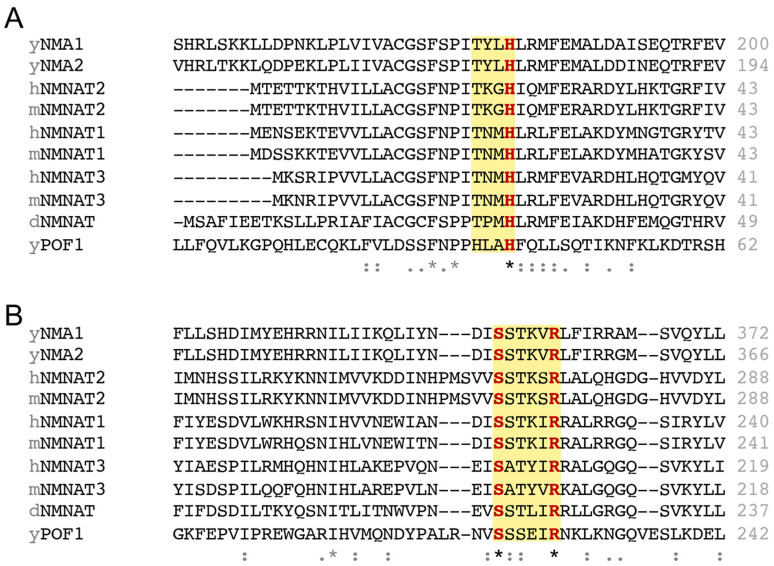
The conserved motifs in NMNATs across different species. Sequence alignment of NMNATs from different species, showing the conserved (**A**) N-terminal “(H/T)XXH” motif required for NAD^+^ synthesis, and (**B**) C-terminal “SXXXXR” motif crucial for chaperone activity. For human NMNATs (hNMNATs), sequences from hNMNAT1 isoform 1, hNMNAT2 isoform 1, and hNMNAT3 isoform 4 are used for the alignment. Multiple sequence alignment across species is conducted using ClustalW. The two motifs are highlighted with a yellow background and the conserved amino acids in the motifs are marked in red. The asterisk (*) indicates that the amino acid is conserved in all the sequences. Double dots (:) indicates that the variant amino acids share similar chemical properties, implying a conserved substitution. Single dot (.) indicates that the variant amino acids share fewer similarities.

NMNATs are highly conserved across species from bacteria to humans. NMNATs from different species share a similar structure, with central parallel β sheets flanked by α helices and a C-terminal domain containing two α helices [46,47]. The central α/β domain harbors the essential motifs for substrate recognition and catalysis. Two conserved regions involved in ATP recognition are found in NMNATs: the “(H/T)XXH” motif in the N-terminal region and the “SXXXXR” motif in the C-terminal region (Figure 2) [48]. The two histidines in the “(H/T)XXH” motif (Figure 2A), respectively, interact with the β phosphate and α phosphate of ATP [49]. Notably, the second histidine is conserved in different species and is essential for catalysis, as a mutation in this residue abolishes NAD^+^ synthesis activity [34,50,51]. The serine and arginine residues in the “SXXXXR” motif (Figure 2B), respectively, interact with the β phosphate and γ phosphate of ATP [49], and surprisingly, this motif is dispensable for NAD^+^ synthesis activity but is required for the chaperone activity of NMNATs [31,35]. Therefore, the “(H/T)XXH” motif is likely sufficient to mediate adequate ATP binding, catalyzing the transfer of the adenylyl moiety of ATP to NMN and NaMN. On the other hand, ATPase activity is often seen in chaperone proteins that hydrolyze ATP to facilitate protein folding or regulate protein assembly. In line with this notion, the “SXXXXR” motif in human NMNAT2 (hNMNAT2) is critical in modulating its ATPase activity [35]. There is no direct evidence showing whether the “(H/T)XXH” motif affects the ATPase activity of NMNATs, which requires further analysis in the future.

**Figure 3:**
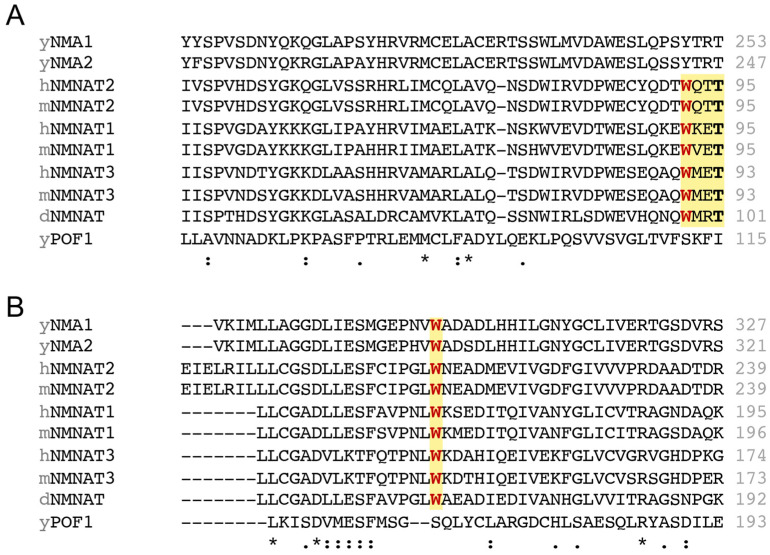
The NMN binding sites in NMNATs across different species. Sequence alignment of NMNATs from different species, showing (**A**) the “WXXT” motif required for NMN binding in *Drosophila* and mammals, and (**B**) the conserved tryptophan residue in all eukaryotic NMNATs except for yeast NMNAT Pof1. For human NMNATs (hNMNATs), sequences from hNMNAT1 isoform 1, hNMNAT2 isoform 1, and hNMNAT3 isoform 4 are used for the alignment. Multiple sequence alignment across species is conducted using ClustalW. The yellow background highlights the conserved amino acids in discussion with the potentially conserved tryptophan marked in red. Conserved threonine shown in the WXXT motif is marked in bold. The asterisk (*) indicates that the amino acid is conserved in all the sequences. Double dots (:) indicates that the variant amino acids share similar chemical properties, implying a conserved substitution. Single dot (.) indicates that the variant amino acids share fewer similarities.

Lastly, the “WXXT” motif is generally considered the NMN binding site and mutating the tryptophan residue in the “WXXT” motif has also been found to disrupt NAD^+^ synthesis activity in different species [34,35]. However, the “WXXT” motif is only found in *Drosophila* and mammals (Figure 3A), not existing in budding yeast. It remains unclear how yeast NMNATs recognize NMN and whether a specific tryptophan plays a role in NMN recognition. On the other hand, some studies have shown that mutating other tryptophan residues outside the “WXXT” motif, such as W169 in hNMNAT1 [52] and W170 in mNMNAT1 [53,54], also abolishes NAD^+^ synthesis activity. Interestingly, this tryptophan residue is also conserved among the NMNATs we examined, except for yeast NMNAT Pof1 (Figure 3B), which raises questions about whether this tryptophan residue could be a putative NMN binding site in yeast and possibly other species. Although several mutagenesis experiments have demonstrated the significance of the tryptophan residue in NAD^+^ synthesis activity, it remains elusive whether a single or multiple tryptophan residues are engaged in NMN recognition. Further experiments are needed to elucidate how different tryptophan residues affect the binding affinity of NMNATs towards NMN and NaMN.

## 4. Subcellular Localization of NMNATs and NAD^+^ Pools

In *Drosophila*, the only NMNAT is alternatively spliced into two major isoforms, thereby resulting in the cytoplasmic NMNAT and the nuclear NMNAT [32]. In mammals, there are three NMNATs with distinct subcellular localizations: NMNAT1 in the nucleus, NMNAT2 in the cytosol and Golgi, and NMNAT3 in the mitochondria. Each human NMNAT contains divergent isoform-specific sequences that are not required for NAD^+^ synthesis activity but regulate the localization of some NMNAT isoforms. The nuclear localization sequence (GRKRKW) is located in the isoform-specific sequence of NMNAT1, and the isoform-specific sequence of NMNAT2 harbors two adjacent cysteines, Cys164 and Cys165, that are required to anchor NMNAT2 to intracellular membranes through palmitoylation [45]. On the other hand, the isoform-specific sequence of NMNAT3 does not affect its mitochondrial localization. Instead, the first 25 amino acids in the N-terminal of NMNAT3 serve as the mitochondrial targeting sequences [45]. The different subcellular localizations of NMNATs contribute to the compartmentalized NAD^+^ pools and influence cellular processes, such as adipocyte differentiation [55] and neuroprotection [32,56].

The compartmentalized NAD^+^ pools are exchangeable and interconnected. In cultured human cells, NAD^+^ concentration in mitochondria is ~230 µM, whereas NAD^+^ concentrations in the nucleus or cytosol are ~100 µM [57]. Many NAD^+^ consuming enzymes have Km values for NAD^+^ around 100 µM [58], indicating that the activity of these enzymes is likely regulated by NAD^+^ concentration. Because the NAD^+^ level in the nucleus is similar to the level in the cytoplasm, NAD^+^ is likely exchangeable between these two pools through the nuclear pores. In line with this notion, deleting NMNAT1 reduces both nuclear and cytoplasmic NAD^+^ concentrations [57,59]. On the other hand, the role of NMNAT3 in maintaining the mitochondrial NAD^+^ pool has been controversial. Deleting NMNAT3 decreases mitochondrial NAD^+^ concentrations in vitro [57], whereas mitochondrial NAD^+^ levels are not altered in NMNAT3-KO mice [60]. Moreover, deletion of NMNAT2 decreases both cytoplasmic and mitochondrial NAD^+^ concentrations [57], implying that cytoplasmic NAD^+^ may be imported into the mitochondria. In fact, mitochondrial NAD^+^ transporters have been found in different species [61,62,63], and SLC25A51 is characterized as the mammalian mitochondrial NAD^+^ transporter [64,65,66]. Several studies have demonstrated that mitochondrial NAD^+^ levels are mainly controlled by the mitochondrial NAD^+^ transporter [64,65,66,67]; however, if this is the case, what is the role of NMNAT3 in the mitochondrial NAD^+^ pool? It is reported that the mitochondrial NAD^+^ pool is connected to other subcellular NAD^+^ pools and buffers NAD^+^ fluctuations in other NAD^+^ pools [67]. Although NMNAT3 does not directly control mitochondrial or total cellular NAD^+^ levels, Hoyland and colleagues showed that deletion of NMNAT3 decreases the mitochondrial NMN level [67]. In addition to synthesizing NAD^+^, NMNATs also catalyze the inverse reaction in which NAD^+^ serves as a substrate to generate ATP and NMN [68]. Moreover, unlike the other two NMNATs that show a lower Km value for NMN (~30 µM), recombinant NMNAT3 has a relatively higher Km value for NMN (209 µM) and a relatively lower Km value for NAD^+^ (130 µM) [68]. It is plausible that NMNAT3 plays a role in buffering NAD^+^ fluctuations by regulating the reversible conversion of NAD^+^ to NMN, which helps preserve NAD^+^ and NAD^+^ equivalents (NMN and ATP) within an optimal range in mitochondria.

Interestingly, the mitochondrial NAD^+^ pool appears to be important in protecting cells from various stresses, including nutrient deprivation, genotoxic stress, and oxidative stress [69,70]. Maintaining a flexible NAD^+^ buffering system in mitochondria is likely helpful for cells to cope with stress promptly. It would be interesting to investigate whether deletion of NMNAT3 compromises cell viability in different stress conditions, which will help strengthen the significance of NMNAT3 and this buffering system. In addition, Hoyland and colleagues carried out experiments in vitro, and most experiments were performed using HEK293 cells. Additional studies in animal models and/or using cell lines that possess a high number of mitochondria, such as cardiac muscle cells and liver cells, may help further confirm these major findings. Further studies to clarify the regulation of this buffering system and examine how this buffering system would affect the activity of NAD^+^-consuming enzymes in different subcellular compartments are warranted.

## 5. The Multifaceted Roles of NMNATs in Neuroprotection

As briefly discussed in the introduction, NMNATs are both NAD^+^ biosynthetic enzymes and neuroprotective factors. In this section, we explicate the three main mechanisms through which NMNATs provide neuroprotection and whether these effects depend on NAD^+^ synthesis activity. To offer a comprehensive understanding of different NMNAT isoforms and NMNATs across different species, we summarize the NMNAT mutants used in neuroprotection studies (Table 1). The roles of SARM1 and NMNATs in neurodegeneration have been thoroughly reviewed [71,72], so this part is excluded from this section.

### 5.1. NMNATs Exert Neuroprotection Through NAD^+^-Dependent Autophagy Boosting

Accumulation of amyloid beta-protein (Aβ) in the brain has been associated with impairments of neurons and cognitive function in patients with Alzheimer’s disease. Autophagy, a self-eating process that removes organelles and proteins through lysosomal degradation, has been reported to alleviate Aβ-induced neurotoxicity [73]. It has been shown that overexpressing hNMNAT1 decreases amyloid aggregates by increasing autophagic activity [74]. Autophagy is typically triggered in response to nutrient starvation through TOR (Target of Rapamycin) signaling but is also regulated by two NAD^+^-consuming enzymes, PARP1 [75,76] and SIRT1 [77,78,79]. For example, PARP1 regulates the nuclear export of AMPK [75], a key factor in initiating autophagy. Meanwhile, SIRT1 physically interacts with Atg5, Atg7, and Atg8, regulating the acetylation of these proteins [77]. It is plausible that overexpressing hNMNAT1 enhances NAD^+^ production in the nucleus, which supports the activity of PARP1 and SIRT1 to promote Aβ degradation via autophagy. On the other hand, PARP1 activity is further increased when PARP1 binds to NMNAT1 [80]. Therefore, overexpressing NMNAT1 may boost autophagic activity through two mechanisms—one dependent on NAD^+^ synthesis and the other on the physical interaction between NMNAT1 and PARP1. To clarify these possibilities, future studies should investigate whether NMNAT1 overexpression raises nuclear NAD^+^ levels. Moreover, studies should also confirm whether the physical interaction between NMNAT1 and PARP1 is enhanced when NMNAT1 is overexpressed. Phosphorylation of NMNAT1 at S136 decreases its binding to PARP1 [80], and further research should examine whether phosphorylated NMNAT1 still promotes autophagy and decreases amyloid aggregates. Lastly, to uncover more mechanistic evidence, using SIRT1 inhibitor or PARP1 inhibitor or in combination will help demonstrate whether SIRT1 and PARP1 are involved in the reduction of amyloid aggregates when NMNAT1 is overexpressed.

Autophagic activity is often measured by levels of proteins involved in autophagy, such as LC3 (Atg8) and p62. A decrease in p62 or an increase in LC3II indicates increased autophagic flux. p62 is a regulatory protein in autophagy that links cargo to the autophagosome [81,82]. Interestingly, p62 forms a ring-like structure around mutant Huntingtin (Htt) aggregates [83], which suggests a role for autophagy in clearing Htt aggregates in Huntington’s Disease. Overexpression of dNMNAT reduces Htt aggregates and Ref(2)P, the *Drosophila* equivalent of human p62 [28], implying that dNMNAT promotes the clearance of Htt aggregates through enhanced autophagic activity. Additionally, in a rat model of glaucoma, a neurodegenerative disease affecting the optic nerve, Kitaoka and colleagues found that rat NMNAT3 alleviates axon degeneration and boosts autophagic flux [84]. This neuroprotective effect of NMNAT3 is blocked by an autophagy inhibitor, confirming that autophagy is involved in NMNAT3-mediated neuroprotection. Although these studies did not directly measure NAD^+^ levels, it was shown that both nicotinamide riboside (NR), an NAD^+^ precursor that effectively raises NAD^+^ levels [85,86,87], and a SIRT1 activator [88] also prevent neurodegeneration by increasing autophagic activity. Overall, these studies suggest that NAD^+^ levels, SIRT1/PARP1 activity, and autophagy are associated in conferring protection in neurological disorders, and that NMNATs may support this protection through NAD^+^ synthesis. Nevertheless, direct evidence addressing whether overexpression of NMNAT3 can increase SIRT1 and/or PARP1 activity is lacking. It is possible that the overexpression of NMNAT3 increases mitochondrial NAD^+^ levels, thereby maintaining the NAD^+^ buffering system to sustain nuclear NAD^+^ levels and support SIRT1 and PARP1 activity. Further studies with enzymatically inactive NMNATs and direct NAD^+^ measurements in different cellular compartments are necessary to clarify the molecular mechanisms as well as the connection between autophagy and NMNATs-mediated neuroprotection.

**Table 1:** A selection of NMNAT mutants in the study of neurological disorders and proteotoxicity.

NMNAT Types	Mutations	Effects	Reference
hNMNAT2	Δ1-100 (removes N-terminus)	Blocks NAD^+^ synthesis No effect on chaperone activity Reduces hAtx-1(82Q) aggregates	[35]
	W92G (NMN binding site)	Blocks NAD^+^ synthesis No effect on chaperone activity Reduces hAtx-1(82Q)/pTau aggregates	[35]
	C164S, C165S (palmitoylation sites)	Increases hNMNAT2 protein stability Increases NAD^+^ synthesis Increases chaperone activity Reduces hAtx-1(82Q)/pTau aggregates	[35]
	Δ269-274 (removes C-terminal ATP binding site)	No effect on NAD^+^ synthesis Reduces chaperone activity (foldase) Fails to reduce hAtx-1(82Q)/pTau aggregates	[35]
	Δ200-303 (removes C-terminus)	No effect on NAD^+^ synthesis Reduces chaperone activity (foldase) Fails to reduce hAtx-1(82Q)/pTau aggregates	[35]
mNMNAT1	H24A (catalytic motif)	Blocks NAD^+^ synthesis No protection against Wallerian degeneration No protection against axotomy-induced Ca^2+^ spike	[89,90]
	R213A, R215A (NLS)	NMNAT1 redistributes from nucleus to cytoplasm Enhances protection against Wallerian degeneration	[91]
mNMNAT3	H22A (catalytic motif)	Blocks NAD^+^ synthesis No effect on chaperone activity	[30]
	KKRK (K55E, K56E, R205E, K206E) (removes “+” charged interface of substrate binding sites)	Blocks NAD^+^ synthesis Reduces chaperone activity	[30]
dNMNAT	H30A (catalytic motif)	Blocks NAD^+^ synthesis Rescues neurodegeneration morphology defect	[34]
	W98G (NMN binding site)	Reduces NAD^+^ synthesis	[34]
	R224A (C-terminal ATP Binding site)	Reduces NAD^+^ synthesis	[34]
	W98G, R224A	Blocks NAD^+^ synthesis Rescues neurodegeneration morphology defect	[34]
	H30A (catalytic motif)	No effect on chaperone activity Reduces hAtx-1(82Q) aggregates	[31]
	W98G, R224A	No effect on chaperone activity Reduces hAtx-1(82Q) aggregates Colocalizes with hAtx-1(82Q) aggregates	[31]
	Δ1-64 (removes N-terminus)	No effect on chaperone activity	[31]
	Δ244-297 (removes C-terminus)	Disrupts chaperone activity	[31]
	ΔCN (removes N- and C-terminus)	Disrupts chaperone activity	[31]
yNMNAT1 (Nma1)	H181A (catalytic motif)	Blocks NAD^+^ synthesis No effect on chaperone activity Reduces polyQ-GFP aggregates Rescues proteotoxicity-caused growth defect	[36]
	Δ375-394 (removes C-terminus; preserves the C-terminal ATP binding site)	No effect on NAD^+^ synthesis Reduces chaperone activity (foldase) Fails to rescue proteotoxicity-caused growth defect	[36]

### 5.2. NMNATs Reduce Protein Aggregates Likely by Acting as Chaperones

Loss of dNMNAT disrupts the structure of photoreceptors in *Drosophila* [34], suggesting a role of dNMNAT in maintaining neuronal integrity. To understand the underlying mechanisms, Zhai and colleagues separated the NAD^+^ synthesis function from dNMNAT by using an enzymatically inactive form of dNMNAT that still preserves the C-terminal ATP binding site but has a mutation in the second histidine of the “(H/T)XXH” motif, which abolishes NAD^+^ synthesis activity [34]. Both wild type (WT) and enzymatically inactive dNMNAT show similar neuroprotective effects in *Drosophila* photoreceptors [34], implying that NAD^+^ could be dispensable in NMNATs-mediated neuroprotection in some circumstances.

Accumulation of misfolded proteins is neurotoxic and has been implicated in several neurodegenerative diseases [92,93]. One approach to managing these diseases is to enhance protein quality control that eliminates misfolded proteins through proteasome degradation. Yeast NMNAT Pof1 has been shown to play a role in protein quality control during ER stress, and Pof1 exhibits ATPase activity similar to that of a chaperone [94]. Additionally, both WT and enzymatically inactive yNMNAT Nma1 restore growth defects caused by proteotoxicity [36,95]. It has also been shown that both chaperones and NMNATs co-localize with protein aggregates, such as human Ataxin-1 with 82 polyQ expansion (hAtx-1[82Q]) [31] and Tau [35]. Furthermore, the Hsp70 chaperone and dNMNAT show similar effects in reducing hAtx-1[82Q] aggregates in a proteasome-dependent manner [31]. These similarities between chaperones and NMNATs suggest that NMNATs may possess chaperone activity to alleviate neurodegenerative disorders caused by protein aggregates. Consistent with this idea, NMNATs display holdase and foldase activities that can prevent protein aggregation and refold misfolded proteins, respectively [31,35]. Moreover, hNMNAT2 displays ATPase activity when complexed with the Hsp90 chaperone protein [35]. Together, these findings suggest that NMNATs may confer neuroprotection through chaperone/ATPase activity.

Tau is a microtubule-associated protein (MAP) that helps stabilize neuronal structures. Abnormally modified Tau, such as phosphorylated Tau (pTau), tends to aggregate into neurofibrillary tangles in the cytoplasm of degenerating neurons, which disrupts neuronal function and leads to neurodegeneration [96]. It is likely that hNMNAT2 works with other chaperones to maintain proteostasis in tauopathies. CHIP (C-terminus of Hsc70 interacting protein; STUB1) is one of the E3 ubiquitin ligases responsible for Tau degradation [97,98]. Phosphorylation of Tau at S416 is shown to weaken its binding to CHIP [99], which likely allows pTau to escape from proteasome degradation. On the other hand, several studies have shown that NMNATs interact with pTau, enhance the recognition of Hsp90 to pTau, and reduce the levels of pTau [29,30,35]. Overexpression of NAD^+^ synthesis-defective hNMNAT2 is shown to reduce pTau [35], suggesting NAD^+^ is dispensable in relieving tauopathies. In comparison, the other mutant form of hNMNAT2, which lacks the C-terminal ATP binding domain (the “SXXXXR” motif) but still has NAD^+^ synthesis activity, shows defective chaperone activity and fails to reduce pTau as well as hAtx-1[82Q] [35]. Therefore, it seems that the chaperone activity of NMNATs can be separated from NAD^+^ synthesis activity, and the chaperone activity alone is sufficient to confer neuroprotection in tauopathies and other proteotoxicities. Although accumulating evidence shows that NMNATs reduce protein aggregates, it remains unclear whether NMNATs only decrease the formation of protein aggregates or also facilitate their degradation. One study shows that both WT and NAD^+^ synthesis-defective dNMNAT reduce the ubiquitination of Tau oligomers [29]. However, it remains unknown how a mutant NMNAT lacking chaperone activity affects the ubiquitination of Tau. Further experiments investigating the ubiquitination of protein aggregates along with a proteasome inhibitor are needed to better characterize the roles of NMNATs as chaperone proteins and elucidate whether NMNATs promote proteasome degradation of specific aggregated proteins.

It remains perplexing how NMNATs dynamically function both as NAD^+^ biosynthetic enzymes and chaperones. Structural analysis reveals that mNMNAT3 uses its positively charged enzymatic pocket to bind to the phosphorylated sites of pTau, NMN, and ATP [30]. Because pTau competes with NMN and ATP for the same binding site, NMNATs may switch roles, acting either as chaperones or as NAD^+^ biosynthetic enzymes, depending on which substrate they bind to. For example, the amount of NMN in mammals is mainly controlled by NAMPT, a rate-limiting enzyme in NAD^+^ synthesis that converts nicotinamide to NMN [13] (Figure 1). During aging, the levels of NAD^+^ and NAMPT decline [100,101], likely limiting NMN production. Therefore, it is plausible that in aging neurons where NMN levels decrease and pTau accumulates, NMNATs function as chaperones to help reduce pTau and other protein aggregates. To test this hypothesis, further studies should examine whether the NAD^+^ synthesis activity of recombinant NMNATs is reduced in the presence of pTau. Additionally, it remains unclear how NMNATs reduce other protein aggregates, such as hAtx-1[82Q]. It is worth investigating whether hAtx-1[82Q] also binds to the enzymatic pocket of NMNATs.

Currently, most studies of the chaperone activity of human NMNATs have been focused on hNMNAT2. Although all hNMNATs possess the C-terminal “SXXXXR” motif (Figure 2), whether hNMNAT1 and hNMNAT3 display chaperone activity and whether they bind to pTau remain unknown. Consequently, a systematic comparison of the three hNMNATs is required for a better understanding of their chaperone activity and their roles in reducing protein aggregates. Additionally, it remains elusive how Hsp90 influences the ATPase activity of hNMNAT2. Since hNMNAT2 complexes with Hsp90 via its C-terminus [35], binding to Hsp90 is likely to induce a conformational alteration. Further analysis examining the structure of NMNAT2-Hsp90 complex may also provide insights into the activation of NMNAT2’s ATPase activity.

### 5.3. NMNATs Enhance Mitochondrial Integrity and Protect Neurons from Injuries

Axon loss is a common feature of neurodegeneration [102]. Wallerian degeneration is triggered by an injury in the distal segment of the axon and serves as a model to study axon degeneration [103]. The NAD^+^ synthesis activity of NMNATs has been implicated in axon degeneration, and NMNAT2 depletion is suggested to be an initiating event in Wallerian degeneration [33]. The C57BL/Wld^S^ mutant mice show a slow Wallerian degeneration phenotype and survive several weeks after transection [104,105,106]. Wld^S^ mutation is a chimeric gene that contains 70 amino acids from the N-terminus of Ube4b (a ubiquitin ligase), unique 18 amino acids generated from gene fusion, and the full-length nuclear mNMNAT1 [107]. After transection, Wld^S^ suppresses Wallerian degeneration and preserves the axon structure [107]. NAD^+^ synthesis activity is at least partially required for Wld^S^-mediated axonal protection because the NAD^+^ synthesis activity of mNMNAT1 increases fourfold in Wld^S^ transgenic mice [107], and mutating the catalytic motif of mNMNAT1 in Wld^S^ reduces its ability to protect against Wallerian degeneration [53,90]. Moreover, targeting Wld^S^ to the cytoplasm by mutating the nuclear localization sequences further bolsters neuroprotection, and the cytoplasmic Wld^S^ is mainly enriched in mitochondria and microsomes [91], suggesting that nuclear localization of mNMNAT1 might limit its protection. Supporting this, Avery and colleagues compared the neuroprotective effects of Wld^S^ along with the three NMNAT isoforms. They found that overexpression of mNMNAT1 exerts less protection against Wallerian degeneration in *Drosophila* neurons, whereas overexpression of mNMNAT3 protects axons in a way similar to Wld^S^ [90]. Another in vivo study shows that neuroprotection is observed only in mNMNAT3-transgenic mice, not in mNMNAT1-transgenic mice [108]. Notably, both NMNAT3 and cytoplasmic Wld^S^ localize to mitochondria, implying that the axonal protection is likely associated with NMNATs’ NAD^+^ synthesis activity in mitochondria. Moreover, as a gain-of-function mutant, it remains possible that Wld^S^ may confer neuroprotection through additional mechanisms in addition to NAD^+^ production. Further studies of the mechanisms underlying the neuroprotection may also help elucidate the cellular functions of NMNATs.

After axotomy, axonal calcium levels rapidly increase within seconds, and the entry of extracellular calcium leads to Wallerian degeneration [109]. Calcium is a signaling molecule with extensive regulation over neurotransmission, synaptic plasticity, and neuronal excitability [110]. Calcium overload is toxic to neurons, and calcium dysregulation has been implicated in neurodegenerative diseases [111,112,113] as well as ischemic stroke [114,115]. Excessive calcium promotes neuronal cell death by activating the calcium-dependent proteases, calpains [116]. Mitochondria possess a calcium buffering capacity that takes up calcium from the cytoplasm, sequesters calcium in the mitochondrial matrix, and maintains calcium homeostasis [117]. However, calcium overload is also harmful to mitochondria as it activates the mitochondrial permeability transition pore, disrupts the inner membrane, and triggers the release of cytochrome c, promoting cell death via a caspase-dependent pathway [118]. NMNATs have been shown to reduce apoptosis and necrosis after hypoxic–ischemic injury [119,120]. Additionally, Wld^S^ and mNMNAT3 have been shown to suppress the axotomy-induced axonal calcium spike by enhancing mitochondrial calcium buffering capacity [89]. Although the mechanisms by which NMNATs improve calcium buffering and reduce cell death are not fully understood, it is plausible that NMNATs modulate NAD^+^ metabolism, coordinate mitochondrial integrity and calcium homeostasis to protect neurons from various stresses and injuries. In line with this notion, a study has shown that overexpressing rabbit NMNAT3 in bone marrow stem cells helps improve mitochondrial function under oxidative stress by restoring ATP, NAD^+^, and mitochondrial membrane potential in a SIRT3-dependent manner [121]. SIRT3 is crucial for mitochondrial integrity, as SIRT3 deacetylates Forkhead box O3 (FOXO3), a transcription factor that regulates genes involved in mitochondrial biogenesis, fission, fusion, and mitophagy [122]. Interestingly, it has been shown that compared with mice, the brain of the naked mole-rat, a hypoxia-tolerant species, is more resistant to calcium overload [123]. While the naked mole-rat has a bigger brain that may contribute to the better calcium buffering capacity, the hyperpolarized mitochondrial membrane potential in the brain is likely another contributing factor. Taken together, it is likely that NMNAT3 increases calcium buffering capacity by supporting mitochondrial NAD^+^ synthesis, which maintains the NAD^+^/NADH ratio, ATP production, and the electrochemical gradient across the inner mitochondrial membrane. Although NMNAT1 and NMNAT2 may not directly contribute to mitochondrial NAD^+^ synthesis, mitochondria can import cytoplasmic NAD^+^ through mitochondrial NAD^+^ transporters. Further studies are needed to examine and clarify the roles of NMNATs in mitochondrial NAD^+^ levels, and how they affect SIRT3 activity as well as mitochondrial integrity in neurological disorders.

## 6. Regulation of NMNATs

**Figure 4:**
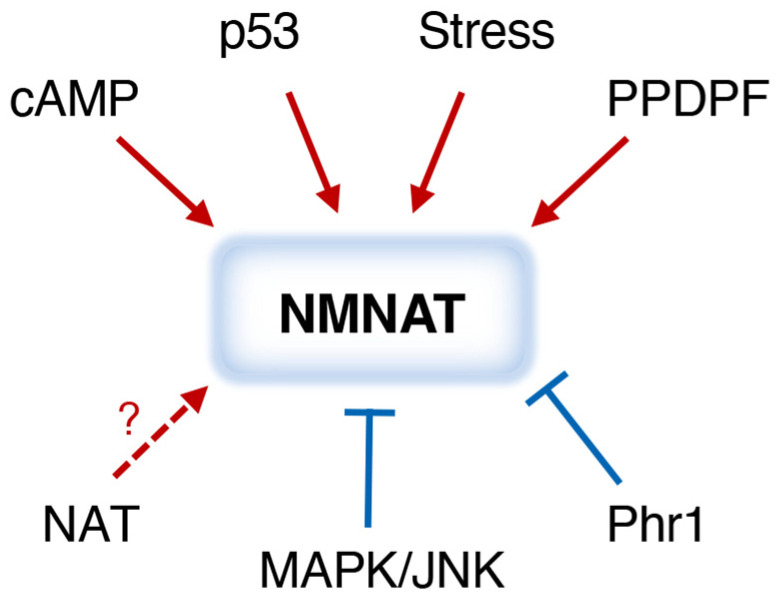
Regulation of NMNATs. A simplified model illustrating factors that regulate NMNATs. cAMP, p53 and stress conditions have been shown to promote NMNAT transcription. NAT complex mediated N-terminal acetylation has been shown to promote NMNAT protein maturation, but this has not been shown in the mammalian system. Phr1 and MAPK/JNK signaling promote the protein turnover of NMNATs. PPDPF is a positive regulator for the NAD^+^ synthesis activity of NMNATs. Dashed line and question mark indicate the connection has not been observed in the mammalian system.

Although the activity and cellular function of NMNATs are extensively studied, less is known about how they are regulated. A simplified model depicting factors that have been associated with the regulation of NMNAT expression and/or activity is shown in Figure 4. So far, most studies have focused on the regulation of mammalian NMNAT2. Both cyclic-AMP (cAMP) response element-binding protein (CREB) and p53 are involved in *NMNAT2* transcription. Two CREB response elements are found in the mouse *NMNAT2* promoter [124]. In line with this, a high-throughput screen has identified positive modulators of NMNAT2, and some of them may increase cAMP concentration to boost NMNAT2 levels via the cAMP-PKA-CREB pathway [125]. Two p53 binding sites are found in the first intron of the human *NMNAT2* gene [126]. It is likely that p53 also regulates the transcription of NMNAT1 and NMNAT3, as p53 can induce the gene expression of all three NMNATs in human cell lines, with a stronger effect on NMNAT2 [126].

Regarding protein regulation, NMNAT2 has the shortest half-life among the three NMNATs, and its turnover is at least in part mediated by proteasome degradation [33]. NMNAT2 is ubiquitinated and recognized by the E3 ubiquitin ligase Phr1/Highwire [127,128]. In addition, Mitogen-Activated Protein Kinase (MAPK) and c-Jun N-terminal kinase (JNK) are also involved in the rapid turnover of mNMNAT2 [129], suggesting extracellular stimuli may activate MAPK-JNK signaling to modulate the protein level of NMNAT2. Interestingly, subcellular localization is another key factor that regulates NMNAT2 stability. NMNAT2 is enriched in numerous membrane compartments and is targeted to membranes through palmitoylation of the two cysteine residues, C164 and C165 [130]. It is shown that mutating these two residues redistributes NMNAT2 into a more diffuse, cytosolic localization and increases its stability [131,132].

Although the regulation of NMNAT1 and NMNAT3 in mammals is relatively less characterized, NMNATs are generally upregulated under stress in different model organisms. In *Drosophila*, *nmnat* is transcriptionally upregulated in stress conditions such as heat shock, hypoxia, and oxidative stress [133]. Heat shock factor (HSF) is the primary transcription factor responsible for this induction, and two HSF-binding sites are located in the promoter of the *Drosophila nmnat* gene [133]. Similarly, the gene expression of NMNAT1 is upregulated in rats and mice under hypoxic conditions [134,135], and NMNAT3 is also increased in neonatal mice experiencing oxygen and blood flow deprivation [120]. Moreover, excessive glutamate is a common factor in excitotoxicity, and glutamate influences NMNAT transcript levels, increasing NMNAT1 and NMNAT2 but decreasing NMNAT3 [136]. Together, these studies indicate that NMNATs are stress response proteins, and different NMNAT isoforms may be involved in distinct stress conditions.

On the other hand, NAD^+^ metabolism has been associated with numerous nutrient-sensing pathways in budding yeast, which include phosphate signaling, amino acid sensing, copper sensing, TOR and cAMP-PKA signaling, and purine metabolism [137,138,139]. Overall, these signaling pathways are connected to the regulation of specific genes in the NR salvage [140], NA-NAM salvage [137], or de novo [141] pathways. Less is known about whether NMNATs are also regulated by these signaling pathways. The findings that mNMNAT2 and dNMNAT are regulated by MAPK-JNK signaling [129] raise questions about whether NMNATs may also be regulated by specific nutrient stresses. While nutrient uptake and utilization are complex in multicellular organisms, the budding yeast *Saccharomyces cerevisiae* could serve as an efficient model organism to study the regulation of NMNATs in different nutrient-deprived conditions. Moreover, it has been shown that yeast NMNATs, Nma1 and Nma2, are regulated through N-terminal acetylation by the NatB complex, affecting protein maturation [142,143]. N-terminal acetylation is a common protein modification that may affect protein–protein interactions, subcellular localization, and protein stability. About 80–90% proteins are N-terminally acetylated [144] by N-terminal acetyltransferases (NATs). Major NAT complexes, including NatA, NatB, and NatC, consist of one catalytic subunit and one auxiliary subunit [145]. Different NATs recognize target proteins based on specific amino acids at the second amino acid following the methionine. For instance, the NatA complex recognizes proteins with alanine, serine, or threonine at the second amino acid while the NatB complex recognizes proteins with aspartic acid, glutamic acid, asparagine, or glutamine at the second amino acid. Yeast cells lacking NatB show an approximately 50% reduction in NMNATs and NAD^+^ levels, and these defects can be rescued by mutating the second amino acid of Nma1, turning it into a substrate of a different NAT complex [142,143]. Similarly to yeast Nma1 and Nma2, mammalian NMNAT1 is a putative target of the NatB complex. On the other hand, mammalian NMNAT2 is a possible N-terminal acetylation target of the NatA complex. Further studies are needed to confirm these assumptions and investigate how N-terminal acetylation affects NMNATs in mammals.

Lastly, the NAD^+^ synthesis activity of NMNATs is regulated by the endogenous pancreatic progenitor cell differentiation and proliferation factor (PPDPF) [146]. In PPDPF-KO mice, NAD^+^ synthesis is defective due to the failure to convert NMN to NAD^+^. While deletion of PPDPF does not affect the mRNA and protein levels of NMNATs, the NAD^+^ synthesis activity of all three NMNATs is decreased. PPDPF is a thiol-disulfide oxidoreductase, and it physically interacts with all three NMNAT isoforms [146]. It is likely that PPDPF regulates the disulfide bond formation crucial for the overall structure of NMNATs and thus modulates the NAD^+^ synthesis activity of NMNATs. However, the mechanistic regulation is uncharacterized. Further studies examining NMNATs’ structure in the presence and absence of PPDPF would help clarify how PPDPF regulates NMNAT enzyme activity and provide insights into future designs of NMNAT activators and inhibitors.

## 7. NMNAT Activators and Inhibitors

NAD^+^ metabolism is an emerging therapeutic target for the treatment and prevention of specific human diseases [8,9,10,11,12]. Since NMNATs are the only enzymes that function in all NAD^+^ biosynthetic pathways (Figure 1), targeting NMNATs may be an efficient strategy for harnessing cellular NAD^+^ levels. Much effort has been made to identify activators and inhibitors of NMNATs, and some of them have been shown to affect the NAD^+^ synthesis activity of NMNATs. In this section, we discuss the properties of NMNAT activators and inhibitors, centering on their impacts on cellular NAD^+^ levels as well as their applications in disease models.

### 7.1. NMNAT Activators

Epigallocatechin gallate (EGCG), a natural compound found in green tea with anti-inflammatory and antioxidant properties, has been reported to enhance the NAD^+^ synthesis activity of purified recombinant NMNATs [68]. EGCG has been implicated in the treatment of cancers [147], neurodegenerative diseases [148], and cardiovascular diseases [149]. Interestingly, EGCG displays the strongest effect on the activity of NMNAT2, with more than two-fold enhancement. In comparison, EGCG activates NMNAT3 by 42%, and NMNAT1 by 10%. EGCG is also shown to increase the protein level of NMNAT2 by three-fold in rat cardiomyocytes, with no effect on NMNAT1 and NMNAT3 [150]. This study suggests EGCG may also enhance NMNAT2 activity by increasing its protein abundance; however, the mechanism remains to be determined. Moreover, it has been reported that EGCG may suppress cardiac hypertrophy [150] and provide neuroprotection [151] in an NMNAT2-dependent manner in murine models, and that EGCG increases NAD^+^ levels both in vitro and in vivo in these studies [150,151].

Despite being an NMNAT2 activator, EGCG is not stable in human intestines, and its absorption rate remains low [152,153]. The poor bioavailability of EGCG hinders its translation into clinical application. To overcome this, Tribble and colleagues used EGCG as a reference compound to design novel small molecules that may increase NMNAT2 activity [151]. Among the ten compounds, nine can increase NAD^+^ levels in specific tissues like the cortex and muscle in mice, and two compounds display significant protective effects against neurodegeneration in retinal ganglion cells [151]. More specifically, they showed that these compounds failed to increase NAD^+^ levels and exert neuroprotective effects when mice were co-treated with FK866, an NAMPT inhibitor that reduces NMN production. EGCG also failed to confer neuroprotective effects in *nmnat2* mutant mice tissues [151]. Therefore, EGCG and EGCG-like compounds appear to increase NAD^+^ from NMN in an NMNAT2-dependent manner. Although observed neuroprotection is likely mediated by the NAD^+^ boosting effect of these compounds, whether and how these compounds directly activate NMNAT2 activity and whether they also increase NMNAT2 abundance remain to be determined. In addition, owing to the dual substrate specificity of NMNATs, future studies should also investigate whether these compounds can increase the NAD^+^ synthesis activity of NMNAT2 when NaMN is used as a substrate. Further studies of these NMNAT2 activators should elucidate the mechanistic association between NAD^+^ levels, NMNAT2 activity, and the observed benefits, such as neuroprotection. Moreover, pharmacodynamics and pharmacokinetics studies are needed to evaluate whether these novel NMNAT2 activators are suitable candidates for clinical application.

To systematically identify more NMNAT2 modulators, Ali and colleagues used the Sigma LOPAC1280 library, containing 1280 pharmacologically active compounds, to screen for both positive and negative regulators that modulate NMNAT2 protein levels [125]. Twenty-four compounds were identified as positive modulators of NMNAT2, and here we name these compounds as “NMNAT2 abundance activators” because the NAD^+^ synthesis activity of NMNAT2 was not examined in the study. Most of their findings agreed with other studies related to NMNAT regulation. For example, they found that some NMNAT2 abundance activators, including 8-Br-cAMP, Ro20–1724, caffeine, the caffeine analog dipropyl-7-methylxanthine, and rolipram, are involved in increasing cAMP signaling. In line with the study showing that two CREB response elements are present in the promoter region of mNMNAT2 [124], these compounds may increase NMNAT2 expression via cAMP-PKA-CREB signaling at the transcription level. Additionally, two MAPK inhibitors were found to increase NMNAT2 expression. Because MAPK signaling promotes the turnover of NMNAT2 [129], MAPK inhibitors are likely to suppress NMNAT2 degradation. Other NMNAT2 abundance activators, such as L-aspartic acid, Bay K8644, L-glutamic acid hydrochloride, pentylenetetrazole, quisqualic acid, and thapsigargin, share the common effect of enhancing neurotransmission, implying that neural activity may also regulate NMNAT2 abundance. Interestingly, NMNAT2 abundance seems to be negatively correlated with neurodegenerative stresses, as *NMNAT2* mRNA levels are decreased in various neurodegenerative diseases [35,154]. The discovery of these NMNAT2 abundance activators may serve as potential therapeutic agents to restore NMNAT2 levels and ameliorate symptoms in neurodegenerative diseases. Supporting this, chronic caffeine treatment is shown to reduce pTau and improve memory function in a tauopathy mouse model [155]. Overall, most NMNAT activators, including EGCG and the compounds described above, seem to increase NMNAT2 protein abundance. It would be interesting to determine whether some of them also directly activate the NAD^+^ synthesis activity of NMNAT2 as in the case of EGCG [68]. As for NMNAT1 and NMNAT3, only EGCG has been reported to increase their NAD^+^ synthesis activity.

### 7.2. NMNAT Inhibitors

Using the Sigma LOPAC1280 library, Ali and colleagues also identified thirteen “NMNAT2 abundance inhibitors” [125]. Among them, cantharidin inhibits protein phosphatase 2A (PP2A), whereas wortmannin inhibits phosphatidylinositol 3-kinase (PI3K). These two compounds have no impact on *NMNAT2* mRNA levels, implying that PP2A and PI3K may regulate NMNAT2 post-transcriptionally. PP2A dephosphorylates key components in MAPK signaling [156,157]. Therefore, inhibition of PP2A by cantharidin is likely to activate MAPK signaling, which promotes NMNAT2 degradation. Other NMNAT2 abundance inhibitors, including bendamustine hydrochloride [158], retinoic acid [159], and PAC-1 [160], are chemotherapy drugs used to treat cancers. How chemotherapy drugs affect NMNAT2 abundance requires further analysis. Nevertheless, NMNAT2 is associated with tumor progression, and its high expression level in solid tumors is likely to promote the occurrence as well as development of tumors [161].

Several NMNAT inhibitors have been identified and tested for their efficacy on NMNATs. For instance, gallotannin, a natural polyphenol found in plants, is a general inhibitor for all three NMNATs [68]. Among the three NMNATs, NMNAT3 is most sensitive to gallotannin inhibition. Gallotannin inhibits the NAD^+^ synthesis activity of NMNAT3 with a half-maximal inhibitory concentration (IC50) of 2 µM. The IC50 values for NMNAT1 and NMNAT2 are 10 µM and 55 µM, respectively. On the other hand, two chemical compounds, 2,3-dibromo-1,4-naphthoquinone (DBNQ) and 2,3-dichloro-1,4-naphthoquinone (DCNQ), are found to potently inhibit NMNAT1, with IC50 values of 0.76 and 1.17 µM [162]. DBNQ is shown to compete with NMN and ATP [162], thereby hampering NMNAT1 activity. Further studies examining the binding between DBNQ and NMNAT1 are needed to confirm whether DBNQ binds to the active site of NMNAT1. The inhibition of DBNQ and DCNQ on the other two NMNATs has not yet been explored. Whether gallotannin, DBNQ, and DCNQ decrease cellular NAD^+^ levels, and whether they affect the expression of NMNATs remain to be studied. Moreover, these inhibitory effects were examined using NMN as a substrate to synthesize NAD^+^. Because NMNATs also convert NaMN to NaAD, additional studies should test how these compounds affect the NAD^+^ synthesis activity of NMNATs in the presence of NaMN.

NAD^+^ analogs represent another promising group of NMNAT inhibitors. NAD^+^ analogs with the addition of a methyl group at the 2′ or 3′-C position of the adenosine moiety, including 2′-MeNAD, 3′-MeNAD, 2′-MeNaAD, and 3′-MeNaAD, have been evaluated as potential inhibitors of the three NMNATs [163]. Among the four, 2′-MeNAD is the most effective NMNAT inhibitor, and 2′-MeNAD selectively and potently inhibits NMNAT2 with an IC50 value of ~20 µM [163]. The inhibitory effects of 2′-MeNAD on NMNAT1 and NMNAT3 are relatively mild. Comparing the inhibitory effects of these four NAD^+^ analogs reveals that the addition of the methyl group at the 2′-C position of the adenosine moiety plays a significant role in inhibiting NMNAT2. The binding between NMNAT2 and 2′-MeNAD is predicted in silico, showing that 2′-MeNAD binds to the active site of NMNAT2 but does not overlap with native NAD^+^ [163]. The other potent NMNAT2 inhibitor is Vacor adenine dinucleotide (VAD), with an IC50 value of 20 µM [164]. VAD exhibits mild inhibition on NMNAT3 with an IC50 value of 463 µM, whereas it does not affect NMNAT1 activity. VAD is synthesized from the pyridine derivative Vacor, which is first converted to Vacor mononucleotide by NAMPT, then adenylated to VAD by NMNAT2. Interestingly, Vacor is shown to quickly deplete NAD^+^ and trigger necrosis in cancer cells by inhibiting NAMPT, NMNAT2, and NAD^+^-dependent dehydrogenases [164]. Currently, NAMPT inhibitors are commonly used to downregulate NAD^+^ synthesis. However, NAMPT inhibitors deplete NAD^+^ slowly (24–48 h) [164,165], whereas VAD treatment is shown to nearly deplete NAD^+^ in 3 h in vitro [164]. The slow and incomplete NAD^+^ depletion by NAMPT inhibitors may stem from the dynamic NAD^+^ biosynthetic routes, as de novo synthesis of QA and salvage of other NAD^+^ precursors, including NA and NR (Figure 1), may still contribute to NAD^+^ production. Moreover, while NAMPT inhibitors repress the conversion of NAM to NMN, the intestinal microbiota possesses the nicotinamidase PncA to convert the accumulated NAM to NA, which is then converted to NAD^+^. The application of Vacor demonstrates that targeting multiple enzymes in NAD^+^ metabolism may reduce NAD^+^ levels more efficiently and promptly, and that NMNATs are promising targets due to their involvement in all NAD^+^ biosynthetic pathways. Taken together, VAD and 2′-MeNAD are by far the most effective and selective NMNAT2 inhibitors. Overall, these studies suggest NMNAT inhibitors are potential anti-cancer drugs. For a more efficient and targeted treatment, it is critical to examine the expression and activity of specific NMNATs that are increased in cancer cells.

## 8. Conclusions and Perspectives

NAD^+^ is the central molecule in cellular physiology and modulating NAD^+^ metabolism is an emerging therapeutic approach for many human diseases. Although this review stems from the additional functions of NMNATs, it is important to note that most of these functions ultimately depend on maintaining NAD^+^ levels and that NMNATs can modulate cellular processes primarily by supporting NAD^+^ homeostasis. Since NMNATs function in all NAD^+^ biosynthetic pathways, targeting NMNATs appears to be a more efficient route to modulate NAD^+^ levels. Enhancing the expression level and activity of NMNATs could be beneficial in the treatment of neurodegenerative diseases due to both their NAD^+^ synthesis activity and chaperone activity. On the other hand, downregulating the expression level or activity of NMNATs could be a strategy for cancer treatment, and NMNAT inhibitors can be used alone or in combination with other cancer drugs to exert synthetic lethality to kill cancer cells. However, to date, only a few NMNAT activators and inhibitors have been identified, and many are still in early stages of research regarding their effects on NMNAT activity and cellular NAD^+^ levels.

Targeting human NMNATs could be challenging because of the existence of three NMNAT isoforms, with distinct subcellular localization and tissue distribution. Although some known NMNAT activators and inhibitors have shown selectivity toward specific isoforms, the IC50 values of these compounds are within the micromolar range, and their effects in disease models require further analysis. Additionally, the pharmacokinetics and toxicities in vivo are not yet determined. More mechanistic studies are needed to evaluate the binding between NMNATs and their activators or inhibitors. For instance, solving the 3D structure of NMNATs in complex with their activators or inhibitors will help optimize the development of these compounds and improve their efficacy. However, the crystal structure of NMNAT2 remains unknown, and the NMNAT2 structure is currently predicted using homology modeling based on the known crystal structures of NMNAT1 and NMNAT3. Advancement in cryogenic-electron microscopy is required to reveal the crystal structure of NMNAT2 in the future. On the other hand, considering that NMNAT activators may increase NAD^+^ levels in neurons to exert neuroprotection, the development of NMNAT activators should evaluate their membrane permeability, which enables these compounds to cross the blood–brain barrier. Lastly, current NMNAT activators and inhibitors are shown to affect NMNATs’ NAD^+^ synthesis activity; it remains unclear whether NMNAT activators and inhibitors also influence the chaperone activity of NMNATs. A potent activator that increases the chaperone activity of NMNATs will be beneficial in reducing protein aggregates in neurodegenerative diseases. Thus, enhancing both NAD^+^ synthesis activity and chaperone activity of NMNATs is likely an approach to strengthen therapeutic effects in neurodegenerative diseases with misfolded proteins.

NAD^+^ precursors supplementation is also a promising regimen to boost NAD^+^ levels and to ameliorate metabolic disorders [8,9,10,11,12]. These precursors are beneficial for increasing or restoring NAD^+^ levels, which helps improve mitochondrial function, energy metabolism, and other cellular processes. NMNATs are likely critical factors in maintaining the beneficial effects of NAD^+^ precursors, as their levels, activity, and subcellular localizations determine whether exogenous NAD^+^ precursors can be efficiently converted into NAD^+^. Moreover, it would be interesting to see whether combining NMNAT activators treatment and NAD^+^ precursor supplementation would have additive and/or synergistic effects. A thorough understanding of how NMNATs are regulated and how cells utilize supplemented NAD^+^ precursors may help optimize therapeutic strategies for NAD^+^ augmentation. To date, although major NAD^+^ biosynthetic enzymes have been extensively studied, how NAD^+^ metabolism is regulated and how its regulation connects to other signaling pathways remain unclear. Furthermore, intracellular compartmentalization of NAD^+^ pools and NAD^+^ intermediates adds complexity to the regulation of NAD^+^ homeostasis. Given the complex interconnection of NAD^+^ biosynthetic pathways and the flexibility of NAD^+^ intermediates, simple model systems such as the budding yeast may serve as efficient tools for the identification of novel NAD^+^ metabolic factors and for studying the mechanistic aspects of the regulation of NAD^+^ metabolism. Although much work remains to be performed, current studies suggest NMNATs are promising targets for modulating NAD^+^ metabolism in the treatment and prevention of human diseases.

## Data Availability

All data are contained within this article.

## Abbreviations

The following abbreviations are used in this manuscript:

3-HA	3-hydroxyanthranilic acid
3-HK	3-hydroxykynurenine
AMPK	AMP activated protein kinase
Aβ	amyloid beta-protein
ATG	Autophagy
cAMP	cyclic-AMP
CD157	ADP-ribosyl cyclase
CD38	NAD^+^ hydrolase
CHIP	C-terminus of Hsc70 interacting protein (STUB1); mediates Tau degradation
CREB	cAMP response element-binding protein
EGCG	epigallocatechin gallate
FK866	NAMPT inhibitor
HAAO	3-hydroxyanthranilic acid 3,4-dioxygenase
Htt	Huntingtin aggregates
HSF	Heat shock factor
IDO	indoleamine 2,3-dioxygenase
JNK	c-Jun N-terminal kinase
KFase	kynurenine formamidase
KMO	kynurenine 3-monooxygenase
KYNU	L-kynurenine hydrolase
L-KYN	L-kynurenine
L-Trp	L-tryptophan
MAPK	mitogen-activated protein kinase
NA	nicotinic acid, nicotinate
NaAD	deamido-NAD^+^
NAD^+^	nicotinamide adenine dinucleotide
NADS	NAD^+^ synthetase
NAM	nicotinamide
NaMN	nicotinic acid mononucleotide
NAMPT	nicotinamide phosphoribosyl transferase
NAPRT	nicotinic acid phosphoribosyl transferase
NAT	N-terminal acetyl transferase
NFK	N-formylkynurenine
NMN	nicotinamide mononucleotide
NMNAT	nicotinamide mononucleotide adenylyl transferase
NR	nicotinamide riboside
NRK	NR kinase; converts NR to NMN
PARP	poly (ADP-ribose) polymerase
PI3K	phosphatidylinositol 3-kinase
PKA	protein kinase A; a nutrient sensing protein kinase
Pnc1	nicotinamidase, yeast
PncA	nicotinamidase, bacterial
PP2A	protein phosphatase 2A
PPDPF	progenitor cell differentiation and proliferation factor
QA	quinolinic acid, quinolinate
QPRT	quinolinate phosphoribosyl transferase
SAM	S-adenosylmethionine
SARM1	NAD^+^ hydrolase
SIRT1	sirtuin 1, NAD^+^-dependent protein deacetylase
SIRT3	sirtuin 3, NAD^+^-dependent protein deacetylase
Tau	a microtubule-associated protein (MAP)
pTau	phosphorylated tau, tends to aggregate in degenerating neurons
TDO	tryptophan 2,3-dioxygenase
TOR	Target of rapamycin; a nutrient sensing protein kinase
Urh1	nucleosidase; Meu1, Urh1, Pnp1 are nucleosidases that convert NR to NAM
Wld^s^	Ube4b-mNMNAT1 fusion protein; suppresses Wallerian degeneration
